# Spanning from the West to East: An Updated Review on Endovascular Treatment of Intracranial Atherosclerotic Disease

**DOI:** 10.14336/AD.2016.0807

**Published:** 2017-04-01

**Authors:** Mohammed Hussain, Neil Datta, Zhe Cheng, David Dornbos, Asif Bashir, Ibrahim Sultan, Tapan Mehta, Faris Shweikeh, Paul Mazaris, Nora Lee, Amre Nouh, Xiaokun Geng, Yuchuan Ding

**Affiliations:** ^1^Department of Neurology, University of Connecticut, Farmington, CT 06030, USA; ^2^Department of Neurology, Luhe Hospital, Capital Medical University, Beijing 101149, China; ^3^Department of Neurological Surgery, The Ohio State University Wexner Medical Center, OH 43210, USA; ^4^Department of Neurosurgery, Seton Hall University, South Orange, NJ 07079, USA; ^5^Department of Cardiothoracic Surgery, University of Pennsylvania, Philadelphia, PA 19104, USA; ^6^Department of Surgery, University of Arizona, Tucson, AZ 85724, USA; ^7^Department of Neurosurgery, Wayne State University School of Medicine, Detroit, MI 48236, USA

**Keywords:** ischemic stroke, randomized controlled trials, intracranial stenting, medical management in ICAD

## Abstract

Ischemic stroke is a major cause of morbidity and mortality, incurring significant cost. Intracranial atherosclerotic disease (ICAD) accounts for 10-15% of ischemic stroke in Western societies, but is an underlying pathology in up to 54% of ischemic strokes in Asian populations. ICAD has largely been treated with medical management, although a few studies have examined outcomes following endovascular treatment. Our objective was to summarize the major trials that have been performed thus far in regard to the endovascular treatment of ICAD and to provide direction for future management of this disease process. Systematic review of the literature from 1966 to 2015, was conducted in regard to intracranial angioplasty and stenting. Studies were analyzed from PubMed, American Heart Association and Society of Neurointerventional Surgery databases. SAMMPRIS and VISSIT are the only randomized controlled trials from which Western guidelines of intracranial stenting have been derived, which have displayed the superiority of medical management. However, pooled reviews of smaller studies and other nonrandomized trials have shown better outcomes with endovascular therapy in select patient subsets, such as intracranial vertebrobasilar stenosis or in the presence of robust collaterals. Suboptimal cases, including longer lesions, bifurcations and significant tortuosity tend to fair better with medical management. Medical management has been shown to be more efficacious with less adverse outcomes than endovascular therapy. However, the majority of studies on endovascular management included a diverse patient population without ideal selection criteria, resulting in higher adverse outcomes. Population analyses and selective utilization of endovascular therapy have shown that the treatment may be superior to other management in select patients.

Although stroke-related death rates have decreased, ischemic stroke remains a major cause of mortality. Nearly 795,000 people suffer a stroke each year, of which approximately 77% (610,000) are first time events [1]. In 2011, 1 in 20 deaths were stroke-related [1]. Apart from mortality, ischemic strokes can lead to severe morbidity and significant expense with direct and indirect cost of stroke totaling $33.6 billion in 2011[1]. Patients with a recurrent stroke had 38% higher costs per patient one year after discharge from index hospitalization when compared to patients with a new stroke [2]. 87% of all strokes are ischemic in nature. Intracranial atherosclerotic disease (ICAD) accounts for 10-54% of all ischemic strokes, amounting to approximately 50,000 events annually [3-5]. From an epidemiological standpoint, higher rates of ICAD are encountered among African American, Asian and Hispanic populations [3-6]. While genetic predilection plays a role in ICAD prevalence in these aforementioned groups, additional factors such as co-morbidities, risk factor profiles and lifestyle are also important determinants [7-9]. From a historical perspective, medical management has been the standard of care for ICAD, with recommendations stemming from the WARSS and WASID randomized trials, which both suggested an overall lack of benefit for warfarin over aspirin for noncardioembolic stroke [10, 11]. In fact, the WASID trial showed that aspirin was associated with an overall lower rate of death or major hemorrhage compared to warfarin, thus favoring aspirin for symptomatic ICAD [10]. Interestingly, in the sub-group analysis, neither medication was effective in patients with severe (70-99% stenosis) symptomatic ICAD [12, 13]. Within this subgroup of patients, an alarmingly high stroke rate, of 18% at 1 year and 19% at 2 years, was observed [13, 14].

While medical management largely consists of anti-platelet therapy and management of co-morbid diseases, endovascular treatments for ICAD include angioplasty with and without stenting[15]. Numerous studies have evaluated the efficacy of endovascular treatment in symptomatic ICAD patients [16-24]; however, the applicability of these studies are limited due to the lack of randomization. While the use of endovascular treatment for acute ischemic stroke has recently been found to be beneficial in the setting of large vessel occlusion within six hours from stroke onset, few randomized trials have looked at the efficacy of endovascular means to treat intracranial stenosis. Our objective was to summarize and comment on the major trials done thus far assessing endovascular treatment for ICAD.

## METHODS

We conducted a literature review of trials that incorporated endovascular treatment modalities for ICAD, including angioplasty, angioplasty with stenting and stenting alone. Our search criteria included trials spanning from 1960 to 2015. The search primarily utilized Pubmed. A review of current evidence based practice guidelines was also obtained from the American Heart Association and Society of Neurointerventional Surgery.

### Endovascular Treatment for ICAD in Western Hemisphere

The utility of endovascular treatment for ICAD began in the 1980s; however, these early studies were largely observational or retrospective single center studies [25, 26]. Following these early results, which demonstrated efficacy in the reduction of stenosis and improvement in blood flow, providers were largely driven to treat ICAD using endovascular modalities [2, 27]. However, results of the first randomized controlled trial (RCT) - the Stenting and Aggressive Medical Management for Prevention of Recurrent Stroke in Intracranial Stenosis (SAMMPRIS) trial had a significant impact in establishing evidence-based guidelines [28]. SAMMPRIS was a multicenter (United States; 50 sites) RCT funded by the National Institute of Neurological Disorders and Stroke (NINDS), aimed at assessing percutaneous transluminal angioplasty and stenting (intervention arm) to prevent recurrent stroke when compared to medical management. Inclusion criteria included TIA or non-disabling stroke within the prior 30 days attributable to severe stenosis (70-99% of the diameter of a major intracranial artery), verified by angiography. The medical management arm consisted of daily aspirin (325 mg) with clopidogrel (75 mg) and management of risk factors, such as hypertension, hypercholesterolemia, diabetes, smoking and obesity. The intervention arm used the self-expanding Wingspan stent (Stryker Neurovascular, Fremont, CA, USA; formerly Boston Scientific Neurovascular) and angioplasty after 75 mg clopidogrel (with or without a loading dose, based on duration of prior clopidogrel dosing). The primary end points of SAMMPRIS included stroke or death within 30 days of the initial incident, stroke or death within 30 days of revascularization, or any ischemic event within 3 years [29]. However, the trial was truncated early when it demonstrated the superior efficacy of medical management. The 30-day rate of mortality or stroke was 14.7% (10.2% ischemic and 4.5% hemorrhagic) in the interventional group compared to 5.8% in the medical management group. On further analysis, the absolute risk reduction from medical management alone was 8.9% at 30 days and 9.0% at 3 years [30]. After 1 year, the 30-day risk of stroke and death and one-year risk of ischemic stroke was 20.0% in the interventional group and 12.2% in the medical management group (4). At the end of the study, medical management compared favorably to interventional treatment (14.9 vs. 23.9%, respectively) with a median follow up of 32 months [31]. As a result, the American Stroke Association recommended maximal medical management for patients with recent stroke or TIA (within 30 days) attributable to severe stenosis (70%-99%) of a major intracranial artery, including aspirin (325 mg daily) and clopidogrel (75 mg daily; for 90 days) *(Class IIb; Level of Evidence B*) [31] . However, these practice guidelines did allow for the investigational use of angioplasty alone with or without placement of a stent for patients with severe stenosis (70%-99%) of a major intracranial artery and actively progressing symptoms after institution of medical management *(Class IIb; Level of Evidence C*) [31]. Interestingly, physician preferences favored endovascular treatment for ICAD following WASID; however, this was short-lived, with a decrease in endovascular treatment seen after SAMMPRIS [32].

The Vitesse Stent Ischemic Therapy (VISSIT) trial was initiated shortly after the start of SAMMPRIS. It was a multicenter (27 sites, 4 international; 3 in China, 1 in Europe) RCT with the goal of evaluating balloon-assisted intracranial stent placement in patients with cerebral or retinal ischemia related to intracranial stenosis in 112 patients [32]. The primary outcome assessed was stroke or transient ischemic attack (TIA), characterized by focal brain or retinal ischemia lasting at least 10 minutes, but resolving within 24 hours, in the same area as the initial insult. Morbidity outcomes were measured by modified Rankin score (MRS) and EuroQol-5D. Patients were evaluated for 1 year following the initial insult. Cardioembolic cases, rapidly worsening (48 hrs) cases, and poor baseline functionality (MRS>3) were excluded. The medical management arm utilized clopidogrel for 90 days, aspirin for one year, statins to reduce LDL <100 mg/dL and blood pressure (BP) control <140 mmHg.

VISSIT demonstrated an increased rate of combined focal brain or retinal stroke or TIA in the endovascular treatment group (24.2% at 30 days, 36.2% at 1 year) compared to the medical management group (9.4% at 30 days, 15.1% at 1 year). Solely evaluating ischemic stroke revealed similar results with the medical management group (5.7% at 30 days, 15.1% at 1 year) faring better than those treated with endovascular stenting (17.2% at 30 days, 34.5% at 1 year). Regarding “hard TIAs,” described as focal brain or retinal ischemia that resolves within 24 hours, a modest benefit was seen with endovascular management (0% at 30 days, 1.7% at 1 year) compared to medical management (3.8% at 30 days, 5.7% at 1 year).

While medical management remains the treatment of choice for most cases involving ICAD, it should be emphasized that careful selection of patients who fail medical management may still benefit from endovascular treatment. When evaluating the outcomes of SAMMPRIS, potential confounding variables may affect the conclusions of the study. From a technical viewpoint, one significant drawback in this study was utilization of ‘over-the-wire’ exchange techniques after balloon angioplasty with subsequent introduction of the stent [29]. This method of wire exchange is known to notoriously introduce thrombotic complications and possible vessel perforation [33]. Other possible factors, such as peri-procedural blood pressure control, peri-operative care in the ICU and antiplatelet resistance, were also not measured in SAMMPRIS, possibly influencing outcomes after endovascular therapy [34, 35]. Subsequent analysis of peri-procedural stroke in SAMMPRIS did show that controlling certain factors, such as clopidogrel loading, higher activated clotting time (ACT) and monitoring wire perforation-related hemorrhages would lead to better outcomes after endovascular treatment [36, 37]. Of note, the initial 2005 FDA approval for the self-expanding Wingspan stent was under the humanitarian device exception grounds for medically refractory patients with TIA or stroke secondary to 50-99% stenosis of a major intracranial artery [4]. This was based on two multicenter registry studies in the USA (the National Institutes of Health [NIH]-sponsored Wingspan registry and the US Wingspan registry) that showed intracranial angioplasty and stenting to have high technical success rates with 30-day stroke rates of 6-9% [22, 23]. In comparison, SAMMPRIS patients were higher risk with 70-99 % intracranial stenosis. Furthermore, patients in the endovascular arm of SAMMPRIS also underwent earlier treatments, with the interquartile between 4 and 16 days after the initial event, potentially leading to decreased plaque stability, increased thrombus formation and a higher incidence of adverse events [38].

In 2014, a systematic review and meta-analysis of studies evaluating stroke recurrence or death in treatment of symptomatic intracranial vertebrobasilar stenosis (IVBS) identified 23 studies [39]. Within these studies, a total of 480 patients with IVBS underwent endovascular treatment compared to 592 patients that underwent medical management. In the final pooled analysis, stroke or death was seen in 14.8 per 100 person-years (95% CI, 9.5 to 20.1) in the medical management arm compared to 8.9 per 100 person-years (95% CI, 6.9 to 11.0) in the endovascular group (95% CI, 1.0 to 1.7) (39). Similarly, a 2009 systematic review of 31 studies and 1177 total procedures evaluating stenting for patients with ICAD was completed [40]. In this study, the mean intracranial stenosis in patients undergoing stenting was 78.7%, and they noted a high technical success rates (median: 96%; interquartile range: 90% to 100%) [40]. Additionally, they found a relatively low rate of complications, including peri-procedural minor or major stroke and mortality, with a median of 7.7 % [40].

Regarding angioplasty alone for ICAD, various retrospective studies have documented reasonable success rates with peri-procedural complication rates ranging between 4-40% [14, 41]. In fact, a 2006 Cochrane review of 79 articles, amounting to 1999 cases, showed overall peri-operative stroke rates of 7.9% and mortality rates of 3.4% [42]. Nevertheless, angioplasty alone carries risk of artery dissection or arterial elastic recoil, contributing to higher restenosis rates [43] .

### Advances in the East

While ICAD accounts for 10-15% of ischemic stroke in Western societies, it is an underlying pathology in up to 54% of ischemic strokes in Asia [2, 44-46]. Multiple studies have looked at endovascular treatment of ICAD in Asia. In South Korea, the reported procedure-related stroke or death rate was 6% in an evaluation of 32 patients with symptomatic middle cerebral artery stenosis treated with intracranial angioplasty [47]. In Hong Kong, a study of 65 ICAD patients were treated with the Wingspan stent system, sustaining a 30-day peri-procedural stroke or death rate of 6.1% with no ischemic or hemorrhagic stroke up to 1 year. Of note, the possibility remains that the risk of recurrent stroke following Wingspan stenting is lower among Asians than Caucasians[48].

Another study was performed in 2013, looking at 77 patients with intracranial atherosclerotic stenosis treated with the Wingspan stent. TIA/stroke and death rates were 5.3% within 30 days, while cumulative TIA/stroke and death rates were 8.1% during a mean 18.9 months’ follow-up period. The design of the Wingspan stent increased procedural difficulty, particularly in tortuous vascular pathways, longer-segment lesions or cases involving arterial bifurcation, which can subsequently increase the risk of in-stent restenosis (ISR) [49]. Similarly, another study investigated the mechanism of procedural failure related to the Wingspan stent, finding higher rates of complications in association with tortuous vascular segments, long (>15 mm) lesions and arterial bifurcations, largely due to the stent’s high radial force and rigidity [50].

China is one of the most populous countries in Asia and a major driver of medical therapies. Over the past 10 years, stroke prevention and treatment has become a national agenda for the Chinese government, and the practice of endovascular procedures has grown rapidly. Unlike the United States, Chinese neurologists play a dominant role in providing interventional therapies, including patients with ICAD and with acute ischemic stroke [29]. ICAD is a common cause of ischemic stroke in China, accounting for up to 54% of cases. Despite the results of SAMMPRIS [28], middle cerebral artery (MCA) stenting continues to be performed routinely in China [29]. One of the reasons underlying this phenomenon is that many Chinese interventionalists thought SAMMPRIS had some inherent design flaws, including neglect of operator experience and treatment in an acute setting following stroke. In addition, basilar artery disease was included, confounding outcome analysis. Use of intracranial stenting is also higher in China due to the fact that the Chinese State Food and Drug Administration (SFDA) has granted permission for both the self-expandable Wingspan and the balloon-assisted Apollo stents (manufactured in Shanghai) for treatment of ICAD [51]. Also, although there were no large RCTs evaluating intracranial stenting in Chinese populations, several cohort studies, case report series and comparative studies have shown positive results.

A 2015 retrospective single-center Chinese study investigated undersized balloon angioplasty followed by placement of the Enterprise stent, demonstrating it to be safe and effective in treating complex symptomatic intracranial atherosclerotic stenosis [52]. This study also demonstrated a higher procedural success rate (100%), lower rate of complications (peri-operative stroke incidence, 9.1%) and better long-term outcome (ISR rate, 6.8%) than published results for the Wingspan stent. It did reiterate, similar to Wingspan stent deployment, the previously mentioned difficulties and higher peri-procedural risks seen with tortuous vascular segments, long lesions or bifurcations.

Another study out of the Beijing Tiantan Hospital revealed a favorable outcome with use of the Wingspan stent in 100 patients with symptomatic intracranial stenosis. Their study demonstrated a low rate of stroke or death (5%) within 30 days (3 ischemic and 2 hemorrhagic strokes). They attributed these beneficial outcomes to the high volumes and increased practitioner experience, suggesting that stenting in high-volume centers may provide a benefit for high-risk patients[53].

These higher success rates were demonstrated in another study as well [38]. The overall technical success rate was 96.3% (highest percentage seen in the sub-population undergoing angioplasty and stenting), with a low 30-day composite stroke, myocardial infarction, or mortality rate of 4.4%. They also argued that the relatively high procedural complication rate in SAMMPRIS was attributable to perforator territory stroke and reperfusion hemorrhage. This study selectively evaluated patients with poor collateral circulation and symptoms of hypoperfusion that would most likely fail medical management. In addition, patients with an acute ischemic stroke within 3 weeks were excluded, thereby allowing time for vessels to stabilize prior to intervention. Lastly, based on arterial access or lesion morphology, different endovascular devices were optimally selected, including balloon mounted stenting, angioplasty alone, and angioplasty with self-expanding stent. Given these strict criteria, the authors were able to demonstrate a high success rate with low rate of complications, stroke, or death.

Although no large RCTs on intracranial stenting for ICAD have been conducted in Asia, several cohort studies, case report series and comparative studies showed positive results. While the current RCTs do not show benefit in regard to endovascular therapy, these other studies have shown benefit in a certain subset of the patient population. Endovascular treatment, established in Western medical theaters, is a rapidly growing specialty in Eastern societies, especially in China. While a RCT is yet needed in this population, Chinese investigators are conducting pivotal trials that could answer questions on the effectiveness, safety and outcomes of endovascular therapies for conditions such as ICAD.

**Table 1:** Comparison of major studies promoting medical management or endovascular therapy.

Medical Management	Endovascular Therapy
**SAMMPRIS** *(RCT)* - Severe stenosis (70-99%) - Multicenter, use of Wingspan stent - Medical management: 14.9% risk of stroke/death at 32 mo - Endovascular: 23.9%	**Abuzinadah et. al, 2014** *(meta-analysis)* - Patients with vertebro-basilar stenosis - Medical: stroke/death in 14.8/100-patient - Endovascular: 8.9/100-patient years
**VISSIT** *(RCT)* - Multicenter, balloon-assisted stent - Medical: 15.1% stroke risk at 1 year - Endovascular: 34.5%	**Feng et. al, 2015** *(retrospective review)* - Single center, balloon-assisted Enterprise stent - 100% technical success rate; 9.1% peri-opertaive stroke risk
	**Jiang et. al, 2011** *(retrospective review)* - Single center, Wingspan stent - 5% stroke/death risk at 30-days
	**Miao et. al, 2015** *(prospective cohort)* - Technical success rate 96.3% - 4.4% stroke/death risk at 30-days

RCT = randomized controlled trial

### Summary and Conclusion

Medical management maintains its supremacy in secondary stroke prevention as demonstrated by SAMMPRIS and VISSIT. Nevertheless, a body of literature also exists, albeit nonrandomized trials, that demonstrates the benefit of endovascular management in a very select subset of the population (Table 1). Further multicenter randomized studies are needed to elucidate and demonstrate the safety and efficacy of endovascular devices in various subsets of ICAD patients.

The SAMMPRIS and VISSIT trials provide a basis for medical management in ICAD as the primary therapy modality, although the therapeutic goals of the medical arm of these trials are exceedingly difficult to achieve. Research from Eastern nations demonstrates promising outcomes for endovascular ICAD therapy, although there are not yet any large randomized clinical trials. Certain subsets of patients, such as those with progressive symptoms despite maximum medical management, vertebro-basilar stenosis, hypoperfusion symptoms or poor collateral circulation, may benefit from endovascular treatment, establishing the importance of patient selection and the need for further trials. The high peri-procedural complications rate experienced in the SAMMPRIS trial was largely due to perforator territory stroke and reperfusion hemorrhage, potentially avoidable with careful patient selection and further technical improvements. Choosing different endovascular approach devices depending on arterial access and lesion morphology can also potentially reduce the risk of perioperative complications. While medical management currently remains the preeminent treatment for ICAD patients following stroke, certain subsets of the population may benefit from endovascular treatment as these therapies continue to evolve.
